# Platelet-lymphocyte ratio as a new predictor of in-hospital mortality in cardiac intensive care unit patients

**DOI:** 10.1038/s41598-021-02686-1

**Published:** 2021-12-08

**Authors:** Guangyao Zhai, Jianlong Wang, Yuyang Liu, Yujie Zhou

**Affiliations:** Beijing AnZhen Hospital, Capital Medical University Affiliated Anzhen Hospital, Beijing, Beijing, 100089 China

**Keywords:** Biomarkers, Diseases, Risk factors

## Abstract

It has been discovered that both inflammation and platelet aggregation could cause crucial effect on the occurrence and development of cardiovascular diseases. As a combination of platelet and lymphocyte, platelet-lymphocyte ratio (PLR) was proved to be correlated with the severity as well as prognosis of cardiovascular diseases. Exploring the relationship between PLR and in-hospital mortality in cardiac intensive care unit (CICU) patients was the purpose of this study. PLR was calculated by dividing platelet count by lymphocyte count. All patients were grouped by PLR quartiles and the primary outcome was in-hospital mortality. The independent effect of PLR was determined by binary logistic regression analysis. The curve in line with overall trend was drawn by local weighted regression (Lowess). Subgroup analysis was used to determine the relationship between PLR and in-hospital mortality in different subgroups. We included 5577 CICU patients. As PLR quartiles increased, in-hospital mortality increased significantly (Quartile 4 vs. Quartile 1: 13.9 vs. 8.3, *P* < 0.001). After adjusting for confounding variables, PLR was proved to be independently associated with increased risk of in-hospital mortality (Quartile 4 vs. Quartile 1: OR 95% CI 1.55, 1.08–2.21, *P* = 0.016, *P* for trend < 0.001). The Lowess curves showed a positive relationship between PLR and in-hospital mortality. The subgroup analysis revealed that patients with low Acute Physiology and Chronic Health Evaluation IV (APACHE IV) or with less comorbidities had higher risk of mortality for PLR. Further, PLR quartiles had positive relation with length of CICU stay (Quartile 4 vs. Quartile 1: 2.7, 1.6–5.2 vs. 2.1, 1.3–3.9, *P* < 0.001), and the length of hospital stay (Quartile 4 vs. Quartile 1: 7.9, 4.6–13.1 vs. 5.8, 3.3–9.8, *P* < 0.001). PLR was independently associated with in-hospital mortality in CICU patients.

## Introduction

The concept of the Coronary Artery Care Unit (CCU) was first proposed in the early 1960s and quickly gained widespread support, which benefitted from rapid technological advances in cardiovascular medicine^1^. At the beginning, the primary purpose of these specialized wards which developer in coronary care established was to reduce mortality in patients with acute myocardial infarction (MI)^2^. Nevertheless, over the past few decades, the CCU's landscape has changed. Mortality of acute coronary syndromes decreased steadily over time^3,4^, while the occurrence of other severe cardiovascular diseases appeared to increase^5^. Because of the greater diversity of diseases among patients admitted to CCU units, the concept of cardiac intensive care unit (CICU) had been used to represent this complex care environment more accurately. It has emerged to provide more targeted services for patients with critical heart diseases at present^6^. Nowadays, CICU has taken on a more important position. And easily accessible prognostic indicators for CICU patients are always welcomed by clinicians.

Previous studies have demonstrated that increased peripheral blood platelet count caused the rise of adverse cardiovascular outcomes, in patients with acute myocardial infarction (AMI), higher platelet count was proved to be associated with mortality and reinfarction within the first year after primary percutaneous intervention (PCI)^7–10^. Low amount of peripheral blood lymphocyte count which reflects inflammatory state, was also confirmed to increase adverse clinical outcomes in patients with cardiovascular diseases, such as congestive heart failure, advanced heart failure, coronary artery disease and unstable angina pectoris^11–15^. As a new prognostic marker, platelet-lymphocyte ratio (PLR) was the combination of the two indexes which provides the concept of platelet aggregation and inflammatory pathways. In clinical practice, elevated PLR was shown to be associated with adverse outcomes. In the area of non-cardiovascular disease, PLR was proved to be an important inflammatory marker that predicted mortality in cancer population^16–18^, critical limb ischemia in peripheral artery disease^19^ and neonate early-onset sepsis^20^. In cardiovascular disease, PLR was proved to be positively correlated with the occurrence of no-reflow after PCI^21^. Moreover, PLR has been proven to be independently associated with long-range survival rate in patients with STEMI and NSTEMI respectively^22,23^. However, no research has demonstrated that how PLR affects patients with severe cardiovascular disease. Thus, investigating the relationship between PLR and in-hospital mortality of patients in CICU was the target of this research.

## Method

### Population selection criteria

**Figure 1:**
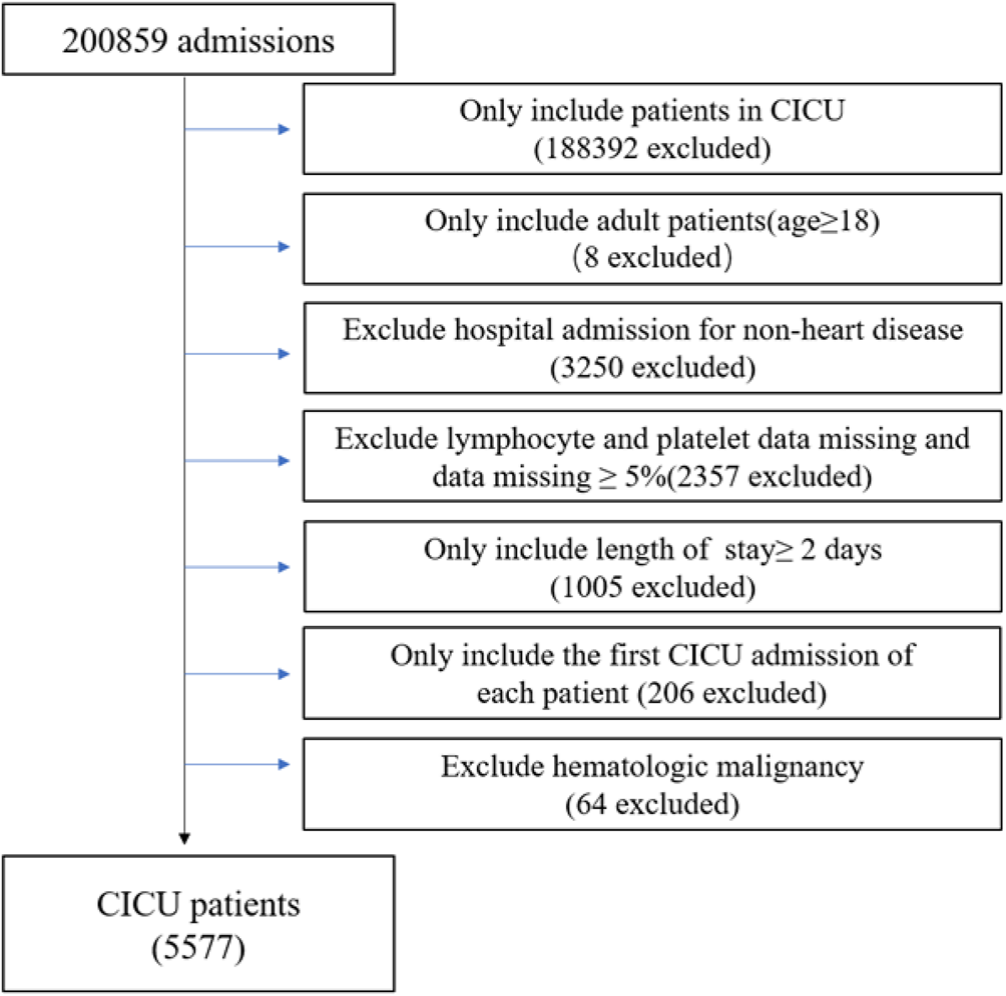
Flow chart of study population. CICU: cardiac intensive care unit.

Patients admitted to CICU were included. Adult patients (≥ 18 years) hospitalized for more than 2 days at their first admission were available. Patients meeting the following criteria were excluded: (1) hospital admission for non-heart disease; (2) lymphocyte and platelet data missing; (3) individual data missing ≥ 5%; (4) hematologic malignancy such as: leukemia and lymphoma. A total of 5577 patients were included (Fig. 1).

### Data extraction

The data used in this study was from eICU Collaborative Research Database^24^, which collected information on 20,859 admissions from 208 hospitals in the United States between 2014 and 2015. The eICU database was made available by Philips Healthcare in partnership with the MIT Laboratory for Computational Physiology. This database is available at: https://doi.org/10.13026/C2WM1R. And the author was approved to access to the database through Protecting Human Research Participants exam (certificate number: 9728458).

Following data were collected: demographics (age, gender and race), vital signs (blood pressure, heart rate, respiration rate, oxygen saturation), body mass index, diagnoses and comorbidities (congestive heart failure, coronary artery disease, acute coronary syndrome, ST-elevation myocardial infarction (STEMI), non-ST-elevation myocardial infarction (NSTEMI), arrhythmias, cardiac arrest, bradycardia, atrial fibrillation, ventricular arrhythmias, atrioventricular block, cardiomyopathy, valve disease, shock, pulmonary embolism, pulmonary hypertension, hypertension, diabetes, hypercholesterolemia, chronic obstructive pulmonary disease (COPD), respiratory failure, chronic kidney disease, acute kidney injury, malignancy, stroke, sepsis), laboratory parameters (white blood cell, lymphocyte, monocyte and neutrophil percentage, red blood cell platelet, hemoglobin, hematocrit, glucose, creatinine, blood nitrogen urea, sodium, potassium, neutrophil to lymphocyte ratio (NLR)), and medication use (antiplatelet, oral anticoagulants, beta-blockers, angiotensin-converting enzyme inhibitor/angiotensin receptor blocker (ACEI/ARB), statin), transfusion (including red blood cell, plasma, platelet), acute physiology score (APS) and Acute Physiology and Chronic Health Evaluation IV (APACHE IV)^25^.

PLR was obtained by dividing platelet count by lymphocyte count. NLR was obtained by dividing neutrophil percentage by lymphocyte percentage. All of the hematological parameters were obtained by the first blood test after admission to CICU within 48 h and measured at the same time.

### Grouping and outcomes

According to PLR quartiles, all enrolled patients were divided into four groups. The primary outcome was in-hospital mortality. Secondary outcomes were length of CICU stay and length of hospital stay.

### Statistical analysis

Normally distributed continuous variables were expressed as mean ± standard deviation (SD) and compared between groups using analysis of variance. Skewed data were expressed as median and interquartile range (IQR) and compared using Kruskal–Wallis test. Categorical variables were expressed as number (percentage) and compared between groups using Chi-square test.

The relationship between PLR and in-hospital mortality was identified by binary logistic regression analysis and the results were expressed as odds ratio (OR) and 95% confidence interval (CI). *P* for trend was calculated. Covariates were selected by statistical analysis and clinical doubt to modulate the outcome. The curves that conformed to the general trend were plotted through local weighted regression (Lowess). Subgroup analysis was used to determine the relationship between PLR and in-hospital mortality in different subgroups, *P* for interaction was calculated. All tests were two-sided, *P* < 0.05 was considered statistically significant. All methods were carried out in accordance with relevant guidelines and regulations. All data analysis were performed by Stata V.15.1.

### Ethical approval

The eICU database was made available by Philips Healthcare in partnership with the MIT Laboratory for Computational Physiology. This study was exempted from institutional review Board approval for the following reasons: (1) retrospective design, which was lack of direct patient intervention; (2) Private certification of reidentification risk conforming to safe harbor standards for security protocols (Cambridge, MA) (HIPAA Certification no. 1031219-2).

### Method statement

All methods were carried out in accordance with relevant guidelines and regulations.

## Result

### Subjects and baseline characteristics

5577 patients admitted to CICU were analyzed (Fig. 1). According to PLR quartiles, all patients were divided into four groups: PLR < 104.8 (n = 1392), 104.8 ≤ PLR < 167.0 (n = 1399), 167.0 ≤ PLR < 271.0 (n = 1392), PLR ≥ 271.0 (n = 1394). The characteristics of different PLR groups were summarized in Table 1, patients with higher PLR had the following characteristics: elder, male, Caucasian, lower systolic, mean and diastolic blood pressure, lower oxygen saturation, and higher heart rate, respiration rate, body mass index. Patients in higher PLR quartiles also tended to present more diagnoses and comorbidities of congestive heart failure, arrhythmias, atrial fibrillation, valve disease, shock, COPD, respiratory failure, chronic kidney disease, acute kidney injury, malignancy, sepsis and less coronary artery disease, acute coronary syndrome, STEMI, NSTEMI, cardiac arrest, ventricular arrhythmias, atrioventricular block, hypertension. Moreover, patients in higher PLR quartiles presented higher white blood cell, neutrophil percentage, platelet, glucose, creatinine, blood nitrogen urea, potassium values and lower lymphocyte, monocyte percentage, red blood cell, hemoglobin, hematocrit, sodium values. Patients in higher PLR quartiles received more oral anticoagulant and less antiplatelet, ACEI/ARB, Beta-blockers, statin treatment. Last but not the least, patients in the highest PLR quartile had highest APS and APACHE IV scores which were used to evaluate the severity of ICU patients and predict their prognosis.

**Table 1:** Characteristics of patients stratified by PLR quartiles.

Characteristics	Total (n = 5577)	Quartiles of PLR				*P* Value
		Quartile 1 (n = 1392) PLR < 104.8	Quartile 2 (n = 1399) 104.8 ≤ PLR < 167.0	Quartile 3 (n = 1392) 167.0 ≤ PLR < 271.0	Quartile 4 (n = 1394) PLR ≥ 271.0	
Age (years)	66.1 ± 15.3	63.2 ± 15.3	66.0 ± 15.3	67.1 ± 15.2	68.3 ± 15.0	< 0.001
Gender, n (%)						0.002
Male	3027 (54.3)	817 (58.7)	739 (52.8)	723 (51.9)	748 (53.7)	
Female	2550 (45.7)	575 (41.3)	660 (47.2)	669 (48.1)	646 (46.3)	
Ethnicity, n (%)						< 0.001
Caucasian	3958 (71.0)	937 (67.3)	952 (68.1)	999 (71.8)	1070 (76.8)	
African American	914 (16.4)	272 (19.5)	241 (17.2)	233 (16.7)	168 (12.0)	
Other	705 (12.6)	183 (13.2)	206 (14.7)	160 (11.5)	156 (11.2)	
Vital signs						
Systolic blood pressure (mmHg)	122.6 ± 19.0	123.2 ± 19.1	123.5 ± 19.7	123.7 ± 19.2	120.1 ± 17.9	< 0.001
Diastolic blood pressure (mmHg)	66.0 ± 11.3	67.3 ± 11.4	66.7 ± 11.6	65.7 ± 11.2	64.2 ± 10.7	< 0.001
Mean blood pressure (mmHg)	82.2 ± 12.9	83.6 ± 12.9	82.8 ± 13.1	82.1 ± 13.2	80.1 ± 12.0	< 0.001
Heart rate (beats/min)	89.2 ± 22.3	85.7 ± 21.8	86.6 ± 21.5	90.5 ± 22.8	94.0 ± 22.1	< 0.001
Respiration rate (beats/min)	21.1 ± 6.3	20.0 ± 6.0	20.8 ± 6.0	21.2 ± 6.2	22.4 ± 6.8	< 0.001
Oxygen saturation (%)	97 (95, 99)	98 (96, 100)	97 (95, 99)	97 (95, 99)	97 (94, 99)	< 0.001
Body mass index (kg/m^2^)	29.3 ± 8.3	29.9 ± 7.9	29.5 ± 8.1	29.6 ± 8.9	28.1 ± 8.3	< 0.001
Diagnoses and comorbidities, n (%)						
Congestive heart failure	1256 (22.5)	261 (18.8)	308 (22.0)	343 (24.6)	344 (24.7)	< 0.001
Coronary artery disease	1853 (33.2)	551 (39.6)	539 (38.5)	407 (29.2)	356 (25.5)	< 0.001
Acute coronary syndrome	1085 (19.5)	362 (26.0)	316 (22.6)	232 (16.7)	175 (12.6)	< 0.001
STEMI	384 (6.9)	149 (10.7)	115 (8.2)	73 (5.2)	47 (3.4)	< 0.001
NSTEMI	403 (7.2)	110 (7.9)	120 (8.6)	89 (6.4)	84 (6.0)	0.027
Arrhythmias	1865 (33.4)	411 (29.5)	444 (31.7)	503 (36.1)	507 (36.4)	< 0.001
Cardiac arrest	407 (7.3)	153 (11.0)	88 (6.3)	93 (6.7)	73 (5.2)	< 0.001
Bradycardia	224 (4.0)	64 (4.6)	59 (4.2)	61 (4.4)	40 (2.9)	0.086
Atrial fibrillation	1117 (20.0)	208 (14.9)	259 (18.5)	320 (23.0)	330 (23.7)	< 0.001
Ventricular arrhythmias	241 (4.3)	82 (5.9)	62 (4.4)	58 (4.2)	39 (2.8)	0.001
Atrioventricular block	119 (2.1)	47 (3.4)	31 (2.2)	23 (1.7)	18 (1.3)	0.001
Cardiomyopathy	365 (6.5)	95 (6.8)	100 (7.2)	96 (6.9)	74 (5.3)	0.189
Valve disease	164 (2.9)	34 (2.4)	38 (2.7)	42 (3.0)	50 (3.6)	0.318
Shock	1718 (30.8)	331 (23.8)	351 (25.1)	425 (30.5)	611 (43.8)	< 0.001
Pulmonary embolism	129 (2.3)	39 (2.8)	30 (2.1)	31 (2.2)	29 (2.1)	0.567
Pulmonary hypertension	69 (1.2)	13 (0.9)	20 (1.4)	17 (1.2)	19 (1.4)	0.647
Hypertension	1689 (30.3)	407 (29.2)	458 (32.7)	441 (31.7)	383 (27.5)	0.011
Diabetes	1133 (20.3)	257 (18.5)	302 (21.6)	297 (21.3)	277 (19.9)	0.144
COPD	633 (11.4)	105 (7.5)	144 (10.3)	170 (12.2)	214 (15.4)	< 0.001
Respiratory failure	1669 (300)	365 (26.2)	360 (25.7)	416 (29.9)	528 (37.9)	< 0.001
Chronic kidney disease	856 (15.4)	163 (11.7)	203 (14.5)	236 (17.0)	254 (18.2)	< 0.001
Acute kidney injury	1031 (18.5)	216 (15.5)	226 (16.2)	276 (19.8)	313 (22.5)	< 0.001
Malignancy	272 (4.9)	38 (2.7)	53 (3.8)	67 (4.8)	114 (8.2)	< 0.001
Stroke	223 (4.0)	58 (4.2)	71 (5.1)	48 (3.5)	46 (3.3)	0.066
Sepsis	1301 (23.3)	207 (14.9)	236 (16.9)	316 (22.7)	542 (38.9)	< 0.001
Laboratory parameters						
White blood cell (10^9^/L)	11.8 ± 6.5	12.0 ± 7.6	11.1 ± 5.7	11.7 ± 6.1	12.3 ± 6.4	< 0.001
Lymphocyte (10^9^/L)	1.6 ± 1.3	2.9 ± 1.7	1.6 ± 0.6	1.2 ± 0.5	0.7 ± 0.3	< 0.001
Monocyte percentage (%)	7.5 ± 3.7	7.8 ± 3.2	8.2 ± 3.4	7.6 ± 3.5	6.6 ± 4.3	< 0.001
Neutrophil percentage (%)	74.7 ± 12.6	64.4 ± 13.2	72.5 ± 9.8	78.2 ± 8.5	83.7 ± 9.6	< 0.001
Red blood cell (10^9^/L)	4.1 ± 0.8	4.2 ± 0.9	4.1 ± 0.8	4.0 ± 0.8	4.0 ± 0.8	< 0.001
Platelet (10^9^/L)	231 ± 102	187 ± 81	214 ± 74	242 ± 93	282 ± 125	< 0.001
Hemoglobin (g/dL)	12.0 ± 2.5	12.7 ± 2.5	12.2 ± 2.4	11.8 ± 2.4	11.2 ± 2.4	< 0.001
Hematocrit (%)	36.4 ± 7.1	38.3 ± 7.2	37.0 ± 6.8	36.0 ± 7.0	34.4 ± 6.8	< 0.001
Glucose (mg/dL)	162.4 ± 98.3	167.7 ± 98.5	156.3 ± 92.3	161.1 ± 95.1	164.9 ± 106.4	0.014
Creatinine (mg/dL)	1.80 ± 1.76	1.65 ± 1.62	1.72 ± 1.71	1.90 ± 1.89	1.93 ± 1.80	< 0.001
Blood nitrogen urea (mg/dL)	30.1 ± 23.4	25.7 ± 19.4	28.0 ± 19.8	32.0 ± 24.9	34.8 ± 27.3	< 0.001
Sodium (mmol/L)	136.9 ± 6.0	137.8 ± 5.3	137.4 ± 5.9	136.8 ± 6.3	135.8 ± 6.4	< 0.001
Potassium (mmol/L)	4.2 ± 0.8	4.2 ± 0.7	4.2 ± 0.8	4.2 ± 0.8	4.3 ± 0	< 0.001
PLR	167.0 (104.8, 271.0)	76.4 (57.0, 90.7)	134.2 (119.3, 148.7)	208.6 (185.9, 236.6)	416.5 (326.8, 584.0)	< 0.001
NLR	6.0 (3.3, 11.7)	2.6 (1.7, 4.0)	4.7 (3.1, 7.2)	7.5 (5.1, 11.3)	15.6 (9.7, 25.5)	< 0.001
Medication use, n (%)						
Antiplatelet	2659 (47.7)	743 (53.4)	711 (50.8)	635 (45.6)	570 (40.9)	< 0.001
Oral anticoagulants	687 (12.3)	141 (10.1)	167 (11.9)	186 (13.4)	193 (13.9)	0.013
Beta-blockers	2446 (43.9)	647 (46.5)	651 (46.5)	591 (42.5)	557 (40.0)	0.001
ACEI/ARB	1490 (26.7)	387 (27.8)	407 (29.1)	385 (27.7)	311 (22.3)	< 0.001
Statin	1717 (30.8)	487 (35.0)	460 (32.9)	399 (28.7)	371 (26.6)	< 0.001
Transfusion	105 (1.9)	23 (1.7)	25 (1.8)	20 (1.4)	37 (2.7)	0.091
APS	41 (28, 57)	36 (25, 56)	38 (26, 53)	42 (30, 56)	46 (34, 61)	< 0.001
APACHE IV	55 (40, 72)	50 (35, 70)	52 (38, 67)	57 (42, 72)	61 (47, 77)	< 0.001

Continuous variables were presented as mean ± SD or median (IQR). Categorical variables were presented as number (percentage).

*PLR* platelet-lymphocyte ratio, *STEMI* ST-elevation myocardial infarction, *NSTEMI* non-ST-elevation myocardial infarction, *COPD* chronic obstructive pulmonary disease, *PLR* platelet-lymphocyte ratio, *NLR* neutrophil to lymphocyte ratio, *ACEI* angiotensin-converting enzyme inhibitor, *ARB* angiotensin receptor blocker, *APS* acute physiology score, *APACHE IV* Acute Physiology and Chronic Health Evaluation IV.

### Association between PLR and outcomes

Overall, in-hospital mortality rate was 10.7%. As PLR quartiles increased, in-hospital mortality increased significantly (Quartile 4 vs. Quartile 1: 13.9 vs. 8.3, *P* < 0.001) (Table 2). In unadjusted logistic regression analysis, there was a positive correlation between PLR and in-hospital mortality (Quartile 4 vs. Quartile 1: OR, 95% CI: 1.77, 1.39–2.25, *P* < 0.001, *P* for trend < 0.001). In model 2, after adjusting for age, gender and ethnicity, higher PLR quartiles were still associated with increased risk of in-hospital mortality (Quartile 4 vs. Quartile 1: OR, 95% CI: 1.63, 1.28–2.09, *P* < 0.001, *P* for trend < 0.001). In model 3, adjusted for more confounding variables, PLR was still independently related to the increased risk of in-hospital mortality (Quartile 4 vs. Quartile 1: OR, 95% CI: 1.55, 1.08–2.21, *P* = 0.016, *P* for trend < 0.001). (Table 3).

**Table 2:** Outcomes of patients stratified by PLR quartiles.

Outcomes	Total (n = 5577)	Quartiles of PLR				*P* Value
		Quartile 1 (n = 1392) PLR < 104.8	Quartile 2 (n = 1399) 104.8 ≤ PLR < 167.0	Quartile 3 (n = 1392) 167.0 ≤ PLR < 271.0	Quartile 4 (n = 1394) PLR ≥ 271.0	
In-hospital mortality, n (%)	597 (10.7)	116 (8.3)	120 (8.6)	168 (12.1)	193 (13.9)	< 0.001
Length of CICU stay (days)	2.3 (1.4, 4.2)	2.1 (1.3, 3.9)	2.2 (1.3, 3.9)	2.5 (1.4, 4.4)	2.7 (1.6, 5.2)	< 0.001
Length of hospital stay (days)	6.3 (3.9, 11.1)	5.8 (3.3, 9.8)	5.8 (3.5, 10.3)	6.4 (4.0, 11.1)	7.9 (4.6, 13.1)	< 0.001

Continuous variables were presented as median (IQR). Categorical variables were presented as number (percentage). *P* values were calculated using Kruskal–Wallis test or Chi-square test to compare differences in outcomes between different PLR quartiles.

*PLR* platelet-lymphocyte ratio, *CICU* cardiac intensive care unit.

**Table 3:** The association between PLR and in-hospital mortality.

	OR (95% CI)	*P* Value	*P* for trend		OR (95% CI)	*P* Value	*P* for trend
**Model 1**			< 0.001	**Model 1**			< 0.001
Quartile 1: PLR < 104.8	Reference			Quintile 1: PLR < 95.5	Reference		
Quartile 2:104.8 ≤ PLR < 167.0	1.03 (0.80–1.35)	0.817		Quintile 2: 95.5 ≤ PLR < 139.0	0.86 (0.64–1.16)	0.320	
Quartile 3:167.0 ≤ PLR < 271.0	1.51 (1.18–1.94)	0.001		Quintile 3:139.0 ≤ PLR < 198.9	1.11 (0.84–1.47)	0.466	
Quartile 4: PLR ≥ 271.0	1.77 (1.39–2.25)	< 0.001		Quintile 4:198.9 ≤ PLR < 314.7	1.35 (1.02–1.77)	0.033	
				Quintile 5: PLR ≥ 314.7	1.74 (1.34–2.27)	< 0.001	
**Model 2**			< 0.001	**Model 2**			< 0.001
Quartile 1: PLR < 104.8	Reference			Quintile 1: PLR < 95.5	Reference		
Quartile 2:104.8 ≤ PLR < 167.0	1.00 (0.76–1.31)	0.999		Quintile 2: 95.5 ≤ PLR < 139.0	0.85 (0.63–1.15)	0.285	
Quartile 3:167.0 ≤ PLR < 271.0	1.43 (1.11–1.84)	0.005		Quintile 3:139.0 ≤ PLR < 198.9	1.06 (0.80–1.41)	0.677	
Quartile 4: PLR ≥ 271.0	1.63 (1.28–2.09)	< 0.001		Quintile 4:198.9 ≤ PLR < 314.7	1.27 (0.97–1.68)	0.086	
				Quintile 5: PLR ≥ 314.7	1.60 (1.23–2.09)	0.001	
**Model 3**			< 0.001	**Model 3**			< 0.001
Quartile 1: PLR < 104.8	Reference			Quintile 1: PLR < 95.5	Reference		
Quartile 2:104.8 ≤ PLR < 167.0	1.49 (1.07–2.07)	0.017		Quintile 2: 95.5 ≤ PLR < 139.0	1.26 (0.87–1.82)	0.214	
Quartile 3:167.0 ≤ PLR < 271.0	1.99 (1.45–2.73)	< 0.001		Quintile 3:139.0 ≤ PLR < 198.9	1.70 (1.19–2.42)	0.003	
Quartile 4: PLR ≥ 271.0	1.55 (1.08–2.21)	0.016		Quintile 4:198.9 ≤ PLR < 314.7	1.62 (1.15–2.30)	0.006	
				Quintile 5: PLR ≥ 314.7	1.47 (1.00–2.17)	0.052	

Models were derived from binary logistic regression analysis. *P* for trend was calculated using binary logistic analysis to determine whether there was a trend when PLR was included as a grouping variable in the model (Quartile 1–4 or Quintile1-5). When PLR was included as a grouping variable in the model, *P* values were calculated using binary logistic analysis to determine whether there was a relationship between PLR quartiles (quintiles) and in-hospital mortality with Quartile1 (Quintile 1) serving as the reference group. When PLR was included as a continuous variable in the model, *P* values were calculated using binary logistic analysis to determine whether there was a relationship between PLR and in-hospital mortality. Model 1: unadjusted. Model 2: adjusted for age, gender, ethnicity. Model 3: adjusted for age, gender, ethnicity, systolic blood pressure, diastolic blood pressure, mean blood pressure, heart rate, body mass index, respiration, coronary artery disease, acute coronary syndrome, congestive heart failure, NSTEMI, cardiac arrest, arrhythmias, atrial fibrillation, ventricular arrhythmias, atrioventricular block, respiratory failure, stroke, malignancy, cardiomyopathy, hypertension, diabetes, white blood cell, red blood cell, hematocrit, blood nitrogen urea, creatinine, sodium, potassium, oral anticoagulants, ACEI/ARB, beta-blockers, statin, transfusion, NLR, APS and APACHE IV.

*PLR* platelet-lymphocyte ratio, *NLR* neutrophil–lymphocyte ratio, *OR* odds ratio, *CI* confidence interval.

**Figure 2:**
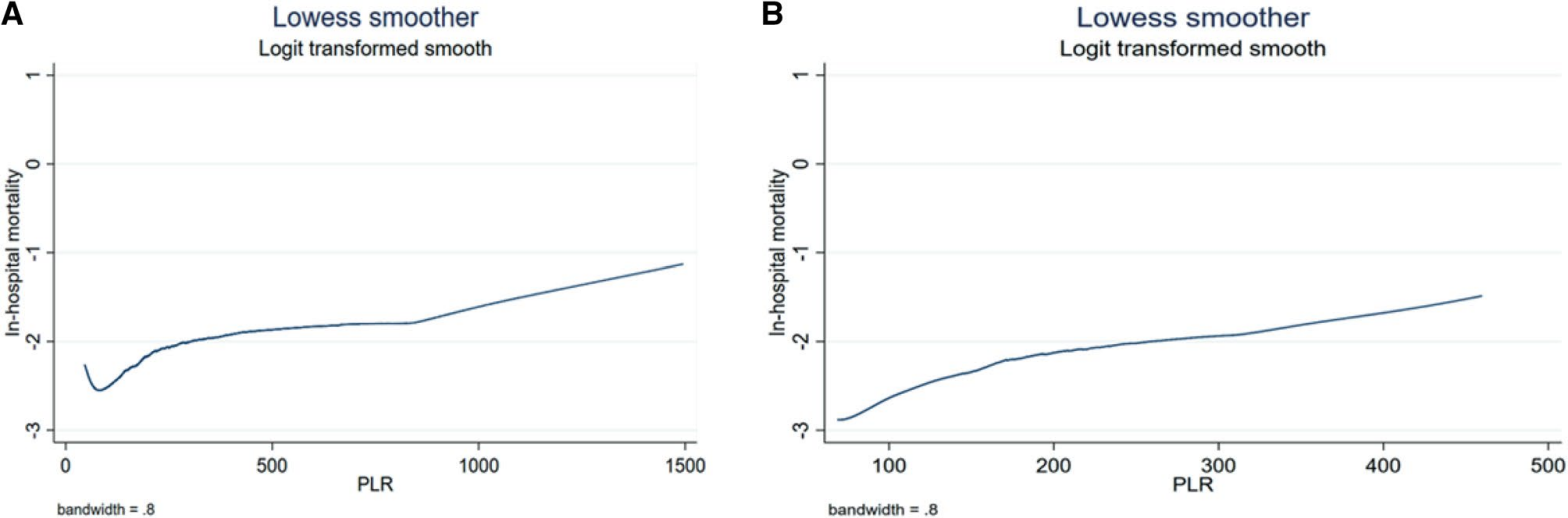
(**A**) Association between PLR and in-hospital mortality presented through Lowess smoothing. (**B**) Association between a range of 10 to 90 percent of PLR and in-hospital mortality presented through Lowess smoothing. Abbreviation: PLR: platelet-lymphocyte ratio.

From Lowess curve in Fig. 2A, we found that mortality was lowest when PLR was about equal to 60. Specifically, when PLR was less than 60, PLR was inversely associated with mortality, while when the PLR was greater than 60, in-hospital mortality increased with the increase of the PLR. Besides, as shown in Fig. 2B, as PLR increased, in-hospital mortality increased with a range of 10 to 90 percent of PLR.

In addition, increased PLR quartiles were associated with prolonged length of CICU stay (Quartile 4 vs. Quartile 1: 2.7, 1.6–5.2 vs. 2.1, 1.3–3.9, *P* < 0.001) and hospital stay (Quartile 4 vs. Quartile 1: 7.9, 4.6–13.1 vs. 5.8, 3.3–9.8, *P* < 0.001) (Table 2).

### Subgroup analysis

No significant interactions were observed in most subgroups. The risk of in-hospital mortality decreased in patients with higher heart rate. Patients with more comorbidities such as coronary artery disease, acute coronary syndrome, hypertension had higher risk of in-hospital mortality for PLR. But patients with cardiac arrest, shock, acute kidney injury had lower risk of in-hospital mortality. Increased risk of in-hospital mortality for PLR was also confirmed in patients with high red blood cell and low glucose, APS, APACHE IV. Besides, patients who received antiplatelet, oral anticoagulants, beta-blocker, ACEI/ARB therapy had higher risk of mortality for PLR (Table 4).

**Table 4:** Subgroup analysis of associations between in-hospital mortality and PLR.

Subgroups	N	Quartile 1	Quartile 2	Quartile 3	Quartile 4	*P* for interaction
**Age (years)**						0.932
< 67	2687	Reference	0.96 (0.64–1.45)	1.47 (1.00–2.15)	1.78 (1.23–2.60)	
≥ 67	2890	Reference	1.02 (0.72–1.46)	1.43 (1.03–2.00)	1.60 (1.16–2.22)	
**Gender**						0.519
Male	3027	Reference	1.12 (0.79–1.59)	1.59 (1.14–2.21)	1.94 (1.41–2.67)	
Female	2550	Reference	0.94 (0.62–1.41)	1.43 (0.98–2.09)	1.57 (1.08–2.29)	
**Ethnicity**						0.677
Caucasian	3958	Reference	1.11 (0.80–1.52)	1.47 (1.09–1.98)	1.84 (1.38–2.45)	
African American	914	Reference	0.64 (0.34–1.19)	1.58 (0.94–2.66)	1.60 (0.91–2.82)	
Other	705	Reference	1.72 (0.71–4.15)	1.77 (0.71–4.45)	1.66 (0.65–4.24)	
**Body mass index (kg/m**^**2**^**)**						0.447
< 27.9	2793	Reference	1.43 (0.97–2.12)	1.76 (1.21–2.57)	1.95 (1.36–2.81)	
≥ 27.9	2784	Reference	0.76 (0.53–1.11)	1.35 (0.96–1.88)	1.69 (1.21–2.36)	
**Systolic blood pressure (mmHg)**						0.257
< 120	2734	Reference	0.99 (0.71–1.38)	1.53 (1.12–2.10)	1.54 (1.14–2.08)	
≥ 120	2843	Reference	1.11 (0.71–1.74)	1.52 (1.00–2.32)	1.96 (1.29–2.99)	
**Diastolic blood pressure (mmHg)**						0.496
< 65	2768	Reference	0.95 (0.68–1.34)	1.32 (0.96–1.80)	1.53 (1.13–2.07)	
≥ 65	2809	Reference	1.14 (0.73–1.77)	1.70 (1.12–2.58)	1.87 (1.23–2.84)	
**Mean blood pressure (mmHg)**						0.907
< 81	2859	Reference	1.10 (0.79–1.55)	1.59 (1.16–2.17)	1.64 (1.21–2.23)	
≥ 81	2718	Reference	0.89 (0.57–1.38)	1.22 (0.80–1.86)	1.57 (1.04–2.39)	
**Heart rate (beats/min)**						0.038
< 87	2744	Reference	0.98 (0.64–1.49)	1.58 (1.06–2.34)	2.08 (1.40–3.08)	
≥ 87	2833	Reference	1.07 (0.75–1.51)	1.36 (0.98–1.88)	1.42 (1.04–1.94)	
**Respiration rate (beats/min)**						0.053
< 20	2313	Reference	1.22 (0.78–1.89)	1.79 (1.17–2.73)	2.13 (1.40–3.24)	
≥ 20	3264	Reference	0.93 (0.67–1.30)	1.33 (0.98–1.81)	1.53 (1.13–2.06)	
**Oxygen saturation (%)**						0.563
< 97	2174	Reference	1.20 (0.78–1.85)	1.69 (1.13–2.53)	2.04 (1.38–3.02)	
≥ 97	3403	Reference	0.94 (0.67–1.32)	1.39 (1.01–1.92)	1.58 (1.15–2.16)	
**Congestive heart failure**						0.661
Yes	1256	Reference	1.39 (0.79–2.44)	1.94 (1.15–3.29)	2.07 (1.23–3.49)	
No	4321	Reference	0.93 (0.69–1.27)	1.37 (1.03–1.83)	1.67 (1.27–2.20)	
**Coronary artery disease**						0.030
Yes	1853	Reference	1.54 (0.94–2.53)	2.26 (1.38–3.70)	2.77 (1.70–4.53)	
No	3724	Reference	0.87 (0.63–1.19)	1.23 (0.92–1.65)	1.41 (1.07–1.87)	
**Acute coronary syndrome**						0.036
Yes	1085	Reference	1.96 (1.09–3.55)	2.58 (1.41–4.72)	3.44 (1.86–6.35)	
No	4492	Reference	0.86 (0.64–1.16)	1.31 (1.00–1.72)	1.51 (1.15–1.96)	
**STEMI**						0.526
Yes	384	Reference	1.33 (0.55–3.18)	1.76 (0.70–4.47)	2.57 (0.97–6.84)	
No	5193	Reference	1.01 (0.76–1.33)	1.49 (1.15–1.93)	1.73 (1.34–2.22)	
**NSTEMI**						0.081
Yes	403	Reference	4.37 (1.42–13.44)	5.37 (1.71–16.83)	6.24 (2.00–19.44)	
No	5174	Reference	0.91 (0.69–1.21)	1.39 (1.07–1.80)	1.63 (1.27–2.10)	
**Arrhythmias**						0.089
Yes	1865	Reference	1.41 (0.85–2.34)	2.19 (1.37–3.49)	2.51 (1.58–3.97)	
No	3712	Reference	0.92 (0.67–1.26)	1.28 (0.95–1.74)	1.52 (1.14–2.04)	
**Cardiac arrest**						0.004
Yes	407	Reference	0.89 (0.52–1.52)	1.44 (0.86–2.42)	1.12 (0.64–1.96)	
No	5170	Reference	1.62 (1.13–2.31)	2.37 (1.69–3.32)	3.21 (2.32–4.44)	
**Bradycardia**						0.162
Yes	224	Reference	7.13 (0.83–61.1)	6.87 (0.80–58.9)	11.12 (1.29–96.18)	
No	5353	Reference	0.98 (0.75–1.29)	1.46 (1.14–1.88)	1.69 (1.32–2.16)	
**Atrial fibrillation**						0.281
Yes	1117	Reference	1.03 (0.54–1.96)	1.91 (1.08–3.38)	2.11 (1.20–3.71)	
No	4460	Reference	1.03 (0.77–1.38)	1.38 (1.04–1.83)	1.65 (1.26–2.17)	
**Ventricular arrhythmias**						0.516
Yes	241	Reference	1.14 (0.47–2.76)	1.85 (0.80–4.27)	1.16 (0.42–3.19)	
No	5336	Reference	1.04 (0.79–1.38)	1.52 (1.17–1.97)	1.86 (1.45–2.40)	
**Atrioventricular block**						0.711
Yes	119	Reference	3.33 (0.57–19.4)	3.38 (0.52–21.79)	2.81 (0.37–21.66)	
No	5458	Reference	1.00 (0.76–1.31)	1.48 (1.15–1.90)	1.74 (1.36–2.22)	
**Cardiomyopathy**						0.464
Yes	365	Reference	1.24 (0.44–3.48)	2.15 (0.83–5.58)	2.43 (0.91–6.53)	
No	5212	Reference	1.02 (0.77–1.34)	1.47 (1.14–1.90)	1.73 (1.35–2.23)	
**Valve disease**						0.425
Yes	164	Reference	0.28 (0.28–2.82)	3.67 (0.93–14.43)	1.97 (0.48–8.03)	
No	5413	Reference	1.06 (0.81–1.38)	1.45 (1.12–1.87)	1.76 (1.37–2.25)	
**Shock**						0.005
Yes	1718	Reference	1.02 (0.67–1.56)	1.29 (0.88–1.91)	1.10 (0.76–1.60)	
No	3859	Reference	1.01 (0.71–1.44)	1.52 (1.10–2.12)	2.05 (1.48–2.84)	
**Pulmonary embolism**						0.746
Yes	129	Reference	3.70 (0.66–20.59)	1.98 (0.31–12.67)	2.13 (0.33–13.69)	
No	5448	Reference	1.00 (0.76–1.31)	1.50 (1.17–1.93)	1.76 (1.37–2.25)	
**Pulmonary hypertension**						0.805
Yes	69	Reference	0.61 (0.75–4.98)	1.18 (0.17–8.33)	0.65 (0.79–5.29)	
No	5508	Reference	1.04 (0.79–1.36)	1.51 (1.18–1.95)	1.79 (1.40–2.29)	
**Hypertension**						0.021
Yes	1689	Reference	2.26 (1.10–4.61)	2.91 (1.45–5.84)	4.08 (2.06–8.09)	
No	3888	Reference	0.92 (0.68–1.23)	1.39 (1.06–1.82)	1.51 (1.16–1.96)	
**Diabetes**						0.415
Yes	1133	Reference	1.10 (0.61–2.00)	1.45 (0.82–2.57)	2.18 (1.26–3.77)	
No	4444	Reference	1.01 (0.75–1.37)	1.53 (1.16–2.01)	1.67 (1.27–2.19)	
**Hypercholesterolemia**						0.369
Yes	411	Reference	0.26 (0.53–1.28)	2.07 (0.79–5.42)	1.83 (0.68–4.94)	
No	5166	Reference	1.09 (0.83–1.43)	1.47 (1.14–1.91)	1.76 (1.37–2.26)	
**COPD**						0.447
Yes	633	Reference	0.63 (0.25–1.62)	1.87 (0.87–4.04)	1.79 (0.85–3.79)	
No	4944	Reference	1.08 (0.82–1.43)	1.44 (1.11–1.88)	1.74 (1.34–2.25)	
**Respiratory failure**						0.061
Yes	1669	Reference	0.98 (0.67–1.43)	1.28 (0.90–1.82)	1.25 (0.90–1.75)	
No	3908	Reference	1.12 (0.75–1.66)	1.66 (1.15–2.41)	1.95 (1.35–2.82)	
**Chronic kidney disease**						0.807
Yes	856	Reference	1.11 (0.59–2.09)	1.15 (0.62–2.11)	1.72 (0.97–3.05)	
No	4721	Reference	1.00 (0.74–1.34)	1.57 (1.19–2.06)	1.71 (1.31–2.25)	
**Acute kidney injury**						0.005
Yes	1031	Reference	0.95 (0.59–1.53)	1.06 (0.68–1.67)	1.03 (0.67–1.60)	
No	4546	Reference	1.06 (0.76–1.47)	1.65 (1.22–2.24)	2.04 (1.52–2.75)	
**Malignancy**						0.017
Yes	272	Reference	0.50 (0.18–1.41)	0.67 (0.26–1.73)	0.56 (0.23–1.34)	
No	5305	Reference	1.07 (0.81–1.41)	1.56 (1.20–2.02)	1.85 (1.44–2.39)	
**Sepsis**						0.063
Yes	1301	Reference	1.20 (0.70–2.06)	1.56 (0.95–2.56)	1.19 (0.74–1.90)	
No	4276	Reference	0.96 (0.70–1.31)	1.37 (1.02–1.84)	1.84 (1.37–2.48)	
**Stroke**						0.882
Yes	223	Reference	2.38 (0.79–7.11)	3.15 (1.01–9.83)	1.90 (0.56–6.44)	
No	5354	Reference	0.97 (0.73–1.27)	1.46 (1.13–1.88)	1.76 (1.38–2.26)	
**Antiplatelet**						0.011
Yes	2659	Reference	1.28 (0.85–1.94)	1.97 (1.33–2.92)	2.52 (1.71–3.71)	
No	2918	Reference	0.86 (0.61–1.23)	1.19 (0.86–1.65)	1.30 (0.95–1.78)	
**Oral anticoagulants**						< 0.001
Yes	687	Reference	5.22 (0.62–43.86)	9.66 (1.24–75.16)	26.79 (3.61–75.16)	
No	4890	Reference	1.01 (0.77–1.32)	1.47 (1.14–1.89)	1.54 (1.20–1.98)	
**Beta-blockers**						0.001
Yes	2446	Reference	2.16 (1.31–3.56)	2.50 (1.52–4.12)	3.73 (2.31–6.02)	
No	3131	Reference	0.73 (0.53–1.02)	1.20 (0.90–1.61)	1.22 (0.91–1.63)	
**ACEI/ARB**						0.001
Yes	1490	Reference	2.32 (1.00–5.36)	3.43 (1.53–7.68)	6.01 (2.74–13.15)	
No	4087	Reference	0.94 (0.71–1.25)	1.36 (1.04–1.78)	1.42 (1.09–1.84)	
**Statin**						0.091
Yes	1717	Reference	1.77 (1.01–3.10)	2.00 (1.14–3.52)	2.91 (1.69–4.99)	
No	3860	Reference	0.86 (0.63–1.17)	1.34 (1.01–1.77)	1.47 (1.11–1.93)	
**White blood cell (10**^**9**^**/L)**						0.232
< 10.3	2761	Reference	0.91 (0.58–1.44)	1.80 (1.19–2.71)	2.49 (1.67–3.73)	
≥ 10.3	2816	Reference	1.15 (0.82–1.60)	1.35 (0.98–1.85)	1.36 (1.00–1.85)	
**Neutrophil percentage (%)**						0.671
< 76	2736	Reference	0.77 (0.52–1.12)	1.25 (0.84–1.86)	1.97 (1.26–3.09)	
≥ 76	2841	Reference	0.88 (0.59–1.33)	0.90 (0.62–1.32)	0.89 (0.62–1.29)	
**Red blood cell (10**^**9**^**/L)**						0.049
< 4.1	2796	Reference	0.98 (0.68–1.41)	1.20 (0.85–1.69)	1.40 (1.01–1.94)	
≥ 4.1	2781	Reference	1.02 (0.69–1.51)	1.79 (1.25–2.57)	2.01 (1.38–2.92)	
**Platelet (10**^**9**^**/L)**						0.151
< 217	2770	Reference	1.18 (0.84–1.66)	1.73 (1.24–2.42)	2.09 (1.48–2.96)	
≥ 217	2807	Reference	0.84 (0.54–1.30)	1.28 (0.87–1.89)	1.51 (1.04–2.20)	
**Hemoglobin (g/dL)**						0.021
< 12.1	2753	Reference	0.81 (0.56–1.18)	1.08 (0.76–1.53)	1.24 (0.89–1.72)	
≥ 12.1	2824	Reference	1.20 (0.82–1.75)	1.90 (1.33–2.72)	2.20 (1.52–3.20)	
**Hematocrit (%)**						0.119
< 36.9	2781	Reference	0.86 (0.58–1.26)	1.14 (0.80–1.62)	1.34 (0.96–1.87)	
≥ 36.9	2796	Reference	1.16 (0.80–1.69)	1.85 (1.30–2.64)	2.12 (1.47–3.08)	
**Glucose (mg/dL)**						< 0.001
< 132	2739	Reference	1.31 (0.84–2.04)	2.44 (1.62–3.68)	2.21 (1.45–3.37)	
≥ 132	2838	Reference	0.94 (0.67–1.33)	1.11 (0.80–1.53)	1.56 (1.16–2.11)	
**Creatinine (mg/dL)**						0.321
< 1.18	2780	Reference	1.29 (0.81–2.07)	1.99 (1.28–3.10)	2.13 (1.37–3.32)	
≥ 1.18	2797	Reference	0.92 (0.66–1.27)	1.25 (0.92–1.69)	1.48 (1.10–1.99)	
**Blood nitrogen urea (mg/dL)**						0.060
< 23	2762	Reference	0.97 (0.65–1.49)	1.55 (1.04–2.29)	1.57 (1.05–2.36)	
≥ 23	2815	Reference	1.00 (0.70–1.42)	1.32 (0.95–1.83)	1.57 (1.15–2.15)	
**Sodium (mmol/L)**						0.632
< 138	2758	Reference	1.11 (0.75–1.67)	1.65 (1.14–2.39)	1.75 (1.22–2.52)	
≥ 138	2819	Reference	0.97 (0.68–1.39)	1.40 (0.99–1.96)	1.83 (1.31–2.57)	
**Potassium (mmol/L)**						0.295
< 4.1	2500	Reference	0.88 (0.58–1.34)	1.33 (0.91–1.95)	1.46 (0.99–2.13)	
≥ 4.1	3077	Reference	1.13 (0.79–1.60)	1.62 (1.16–2.25)	1.94 (1.41–2.67)	
**Transfusion**						0.128
Yes	105	Reference	4.19 (0.43–40.62)	2.44 (0.20–29.19)	9.31 (1.11–77.88)	
No	5472	Reference	1.01 (0.77–1.32)	1.50 (1.17–1.93)	1.69 (1.32–2.16)	
**APS**						< 0.001
< 41	2729	Reference	2.52 (1.15–5.51)	4.61 (2.19–9.70)	6.16 (2.93–12.92)	
≥ 41	2848	Reference	0.87 (0.65–1.18)	1.04 (0.79–1.38)	1.04 (0.79–1.36)	
**APACHE IV**						< 0.001
< 55	2747	Reference	2.55 (1.17–5.57)	3.96 (1.85–8.48)	6.61 (3.16–13.81)	
≥ 55	2830	Reference	0.85 (0.63–1.15)	1.02 (0.77–1.35)	0.99 (0.76–1.30)	

Binary logistic regression analysis was used and results were presented as OR (odds ratio) and 95% CI (confidence interval). *P* for interaction was calculated using binary logistic analysis to determine whether there is interaction between different subgroups and PLR quartiles.

*STEMI* ST-elevation myocardial infarction, *NSTEMI* non-ST-elevation myocardial infarction, *COPD* chronic obstructive pulmonary disease, *PLR* platelet-lymphocyte ratio, *ACEI* angiotensin-converting enzyme inhibitor, *ARB* angiotensin receptor blocker, *APS* acute physiology score, *APACHE IV* Acute Physiology and Chronic Health Evaluation IV.

## Discussion

No studies have been conducted on CICU patients who are more in need of a simple prognostic factor such as PLR. This is the first study to explore the role of PLR in patients with severe cardiovascular disease, which will provide a clinical basis for the application of PLR in CICU patients. This study confirmed the relationship between PLR and in-hospital mortality in CICU patients. (1) As PLR quartiles increased, in-hospital mortality increased significantly. And after adjusting for possible confounding variables, PLR was still independently associated with in-hospital mortality. (2) The Lowess curves presented a positive relationship between PLR and in-hospital mortality. (3) Significant interactions were observed in several subgroups. (4) Length of CICU and hospital stay were prolonged as PLR increased.

In the previous study, inflammation has been proven to be strongly associated with development and prognosis of cardiovascular disease^26^. And there was evidence that lymphocytes played a key role in the regulation of inflammatory response at all levels during atherosclerosis. During the systemic inflammatory response, the lymphocyte count was proved to decrease because of increased lymphocyte apoptosis^27^. This may explain the underlying mechanism for the diagnostic and prognostic validity of low lymphocyte count in patients with acute coronary syndrome and stable coronary artery disease (CAD), respectively^14,28^. The prethrombotic state is caused by increased megakaryocyte series proliferation and relative thrombocytosis, which reflects the body's persistent inflammatory state^7^. Moreover, some studies have demonstrated an increase in the incidence of adverse events as the platelet count increased^7–10^. Patients with higher platelet count corresponded to worse outcomes in ACS^29^. The reason may be that elevated levels of platelet mononuclear aggregation (PMA) in the blood of patients with coronary heart disease, which correlated with plaque stability^30,31^. And high PMA levels in patients with NSTEMI have been proven to increase the risk of adverse outcomes^32^. As a member of systemic inflammatory response family, PLR is the combination of platelet and lymphocyte, which represents the situation of aggregation and inflammatory pathways, and is able to amplify changes in these two indicators, especially in cases where some clinicians tend to overlook such changes, such as when the indicator values are near the upper or lower limits of normal. Paying attention to PLR in clinical practice and improving the level of nursing and monitoring may improve the prognosis and reduce mortality.

As an available indicator, PLR has already been proven to be associated with severity and prognosis of cardiovascular disease. An observational study which enrolled 619 patients with NSTEMI confirmed that high PLR could independently predict the increased of long-term mortality^22^. And previous study included 636 patients with ST-elevated acute myocardial infarction showed that PLR was an independent predictor of cardiovascular mortality^23^. Moreover, PLR was proved to be a conventional risk factor in predicting severe atherosclerosis, and independently associated with increased Gensini score^33^. Besides, it was showed that high preoperative PLR level increased the incidence of no-reflow in patients after PCI^20^.

Our research reached a similar conclusion that increased PLR was independently associated with in-hospital mortality in CICU patients, providing evidence for the use of PLR in patients with severe cardiovascular disease. In the subgroups of congestive heart failure, coronary artery disease, valvular disease, cardiomyopathy, arrhythmias, and shock, the same conclusion could be reached. These diseases almost covered most diseases in CICU, which confirmed the reliability of PLR application in CICU patients.

Through the Lowess curves, it has been demonstrated that when PLR was lower than 60, the mortality rate decreased with the increase of PLR, which suggested that we should be flexible when using PLR to judge the disease condition of CICU patients. When the PLR is very small, consideration should be given that whether the patient has other comorbidities that may increase mortality, such as diseases of the blood system. In patients with idiopathic thrombocytopenia, the platelet count is less than 100*10^9^/L, and even less than 10*10^9^/L in severe cases, resulting in an extremely small PLR value. In this study, we only excluded patients with hematologic malignancies from hematologic diseases. And due to retrospective studies, we could not rule out the possibility of missed diagnosis.

Therefore, when applying PLR in clinical judgment, it is unreasonable to think that the smaller the PLR, the better, and it is necessary to define a threshold value. The value of 10–90%PLR was set as the reference range, and the Lowess curve showed that the mortality rate increased with the increase of PLR, that is, there was no inflection point. In this way, a more reasonable reference range for PLR was 69–460. When the PLR is below 69, the patients should be considered for other comorbidities that may increase mortality. When the PLR is greater than 460, we need to be aware that the patients' condition may be severe with a higher mortality rate.

In addition, as PLR quartiles increased, the length of hospital stay and the length of hospital stay significantly increased, which brought the psychological, physical, and financial burden on patients. Therefore, more attention should be paid to inexpensive, easily accessible indicators like PLR, which is more cost-effective, especially in some cases that more complex score could not be calculated, for example, the patient is unable to undergo complex examination or the patient is in a remote area without the condition to do so.

Other classic prognostic markers, such as neutrophil–lymphocyte ratio (NLR), were also proved to be associated with the prognosis of patients with severe cardiovascular disease^34^. In contrast, few studies on PLR were conducted. In our study, we explored the relationship between PLR and in-hospital mortality in CICU patients and used the receiver operating characteristic (ROC) analysis to assess the ability of NLR and PLR in predicting the incidence of death. In comparison with PLR, the area under the ROC curve (AUC) of NLR was larger (NLR vs. PLR: 0.63 vs. 0.60), which suggested that NLR has a better ability to indicate the risk of in -hospital mortality. Meanwhile, we added NLR to Model 3, and the results indicated that PLR was related to in-hospital mortality independently of NLR. The purpose of our study was not to prove that PLR was the best predictor, but to improve the theoretical basis of PLR application in CICU patients. In clinical practice, the judgment of a patient's condition cannot only rely on a single indicator. We can make a clearer judgment of the patient's condition by using all the indicators we have already known, and through the exploration of these simple prognostic indicators, we will be able to use these indicators to build a prognostic model of CICU patients and confirm it in prospective studies.

Independent association between PLR and in-hospital mortality in CICU patients was proved in this study, which is convenient for clinical use. The multi-center and large sample size made the conclusion more reliable. However, this study also had some limitations. First of all, bias is inevitable due to the retrospective study. Secondly some important indexes can’t be collected such as left ventricular ejection fraction, C-reactive protein, cholesterol. Generally speaking, the accuracy of the model is determined by the variables in the model, and the accuracy of the model in this study is affected to a certain extent due to the lack of the above variables. This will be improved in further research. The inability to dynamically analyze PLR was also the limitation.

## Conclusion

To sum up, the results indicated that PLR was an independent predictor of CICU patient mortality in hospital. The in-hospital mortality rate increased significantly as PLR quartiles increased. Further, high PLR was related to prolonged CICU and hospital stay length. And patients with low APACHE IV or with less comorbidities had higher risk of mortality for PLR.

## Supplementary Information

Supplementary Information.

## Data Availability

The data used in this study was from eICU Collaborative Research Database^24^, which is available at: https://doi.org/10.13026/C2WM1R. The author was approved to access to the database through Protecting Human Research Participants exam (certificate number: 9728458).
